# Turning the fate of reprogramming cells from retinal disorder to regeneration by Pax6 in newts

**DOI:** 10.1038/srep33761

**Published:** 2016-09-19

**Authors:** Martin Miguel Casco-Robles, Md Rafiqul Islam, Wataru Inami, Hibiki Vincent Tanaka, Ailidana Kunahong, Hirofumi Yasumuro, Shiori Hanzawa, Roman Martin Casco-Robles, Fubito Toyama, Fumiaki Maruo, Chikafumi Chiba

**Affiliations:** 1 Faculty of Life and Environmental Sciences, University of Tsukuba, Tennodai 1-1-1, Tsukuba, Ibaraki 305-8572, Japan; 2 Department of Genetic Engineering and Biotechnology, University of Chittagong, Chittagong-4331, Bangladesh; 3 Graduate School of Life and Environmental Sciences, University of Tsukuba, Tennodai 1-1-1, Tsukuba, Ibaraki 305-8572, Japan; 4 Graduate School of Engineering, Utsunomiya University, Yoto 7-1-2, Utsunomiya, Tochigi 321-8585, Japan

## Abstract

The newt, a urodele amphibian, has an outstanding ability– even as an adult –to regenerate a functional retina through reprogramming and proliferation of the retinal pigment epithelium (RPE) cells, even though the neural retina is completely removed from the eye by surgery. It remains unknown how the newt invented such a superior mechanism. Here we show that disability of RPE cells to regenerate the retina brings about a symptom of proliferative vitreoretinopathy (PVR), even in the newt. When Pax6, a transcription factor that is re-expressed in reprogramming RPE cells, is knocked down in transgenic juvenile newts, these cells proliferate but eventually give rise to cell aggregates that uniformly express alpha smooth muscle actin, Vimentin and N-cadherin, the markers of myofibroblasts which are a major component of the sub-/epi-retinal membranes in PVR. Our current study demonstrates that Pax6 is an essential factor that directs the fate of reprogramming RPE cells toward the retinal regeneration. The newt may have evolved the ability of retinal regeneration by modifying a mechanism that underlies the RPE-mediated retinal disorders.

In contrast to other vertebrates including humans, the newt (a group of urodele amphibians classified into the family of *Salamandridae*) can regenerate, even as an adult, an entire retina from retinal pigment epithelium (RPE) cells^1^,^2^. In adult vertebrate eyes, the RPE is a highly differentiated monolayer-cell-sheet laminating the back of the neural retina (NR) and functions as a partner of the NR for vision. Mature RPE cells, as a rule, do not proliferate under physiological conditions. In the adult newt, RPE cells in the intact eye are also mitotically inactive, but when the NR is removed from the eye by surgery (retinectomy), RPE cells lose their epithelial characteristics and detach from each other as well as from the basement membrane (Bruch’s membrane), giving rise to the aggregates of mesenchymal-like cells with multipotency, named RPE stem cells (RPESCs), in the vitreous cavity. RPESCs are subsequently divided into two cell populations which undergo proliferation, so that they can differentiate in the correct polarity into two epithelial layers of progenitor cells (named pro-NR and pro-RPE layers) that eventually regenerate new functional NR and RPE, respectively^3^.

It remains unknown how such a sophisticated mechanism for retinal regeneration evolved in the newt. It would be difficult to understand this mechanism solely by the mechanisms underlying RPE-to-NR transdifferentiation which can be induced in a restricted time frame during embryonic development in amniotes. In these cases, the RPE does not lose its epithelial characteristics, but directly switches into the neuroepithelium while preserving its original polarity, giving rise to the NR with inverse polarity while losing the RPE^2^,^3^,^4^. On the other hand, our recent studies revealed a similarity in early behaviour of RPE cells between adult newt retinal regeneration and human retinal disorders such as proliferative vitreoretinopathy (PVR)^3^,^4^. In PVR, when the NR suffers a wound from a traumatic injury, RPE cells – as in the newt – start to lose their epithelial characteristics while acquiring the ability to migrate and proliferate. However, unlike in the newt, these cells eventually transform into myofibroblasts, a major component of both the epi- and sub-retinal membranes which close the wound of the NR, but finally withdraw the NR by contraction, leading to a loss of vision. In this process of transformation (classified as the epithelial-mesenchymal transition, or EMT), it has been suggested that RPE cells pass through a multipotent state^4^. Such multipotent RPE cells in humans, which were also named RPESCs, are regarded as the cells corresponding to the newt RPESCs^3^,^4^.

Perhaps, in the newt something may have happened in early processes of retinal disorders like PVR during evolution, so that the fate of RPESCs was directed toward retinal regeneration. If this is the case, when retinal regeneration in the newt is impaired in early processes, symptoms of PVR would become apparent. In this study, we examined this hypothesis. For this, we created for the first time a transgenic newt enabling RPE-targeted gene regulation and successfully hindered retinal regeneration by knocking down the expression of Pax6 in RPESCs. Pax6 is a transcription factor which is re-expressed in RPESCs and then up-regulated in the pro-NR layer while down-regulated in the pro-RPE layer^3^.

## Results

### Conditional RPE-targeted gene knockdown system

We created transgenic newts (*Cynops pyrrhogaster*) to specifically express shRNA as well as a fluorescent reporter protein mCherry in both mature RPE cells and cells that originate from those RPE cells after retinectomy. These animals carried DNA encoding either control shRNA (*C-shRNA*) or Pax6 shRNA (*Pax6 shRNA-2*), which was located between the stop codon and the polyA terminal in the *mCherry* gene (Supplementary Fig. 1). These shRNAs were examined in our previous study^3^, and *Pax6 shRNA-2* proved to effectively inhibit Pax6 expression in this species. In this study, we used juvenile newts around 7 months old (1–2 months after metamorphosis). They were incubated at the swimming larva stage (St. 58–59; around 4 months old) in a rearing solution containing 4 μM (Z)-4-Hydroxytamoxifen (4-OHT) for 48 h. In this condition, Cre-mediated recombination leading to mCherry-shRNA expression was almost restricted in the central area of the RPE in metamorphosed newts (Supplementary Figs 2 and 3): in juvenile newts around 7 months old, more than 70% of the cells in the central RPE (45°–135°) and less than 30% of the cells in the peripheral RPE were mCherry+. In addition, all transgenic newts used in this study displayed no abnormalities in eye development or in NR/RPE morphology.

### Retinal regeneration in the control condition

**Figure 1:**
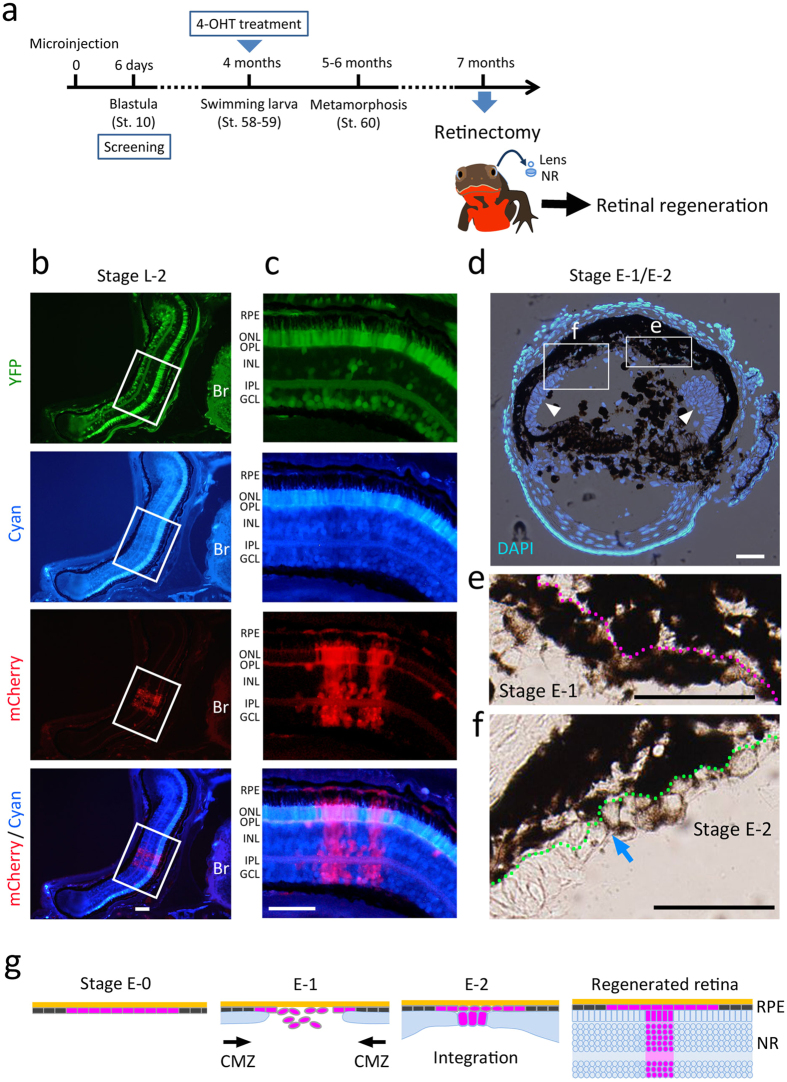
Retinal regeneration in control condition. (**a**) Time schedule of experiments. One-cell stage embryos were microinjected with a solution containing both a Cre driver construct (RPE65 > CreER^T2^-CAGGs > YFP) and a loxP reporter/shRNA construct (CAGGs > [AmCyan]mCherry-shRNA). On the sixth day after injection, blastula embryos (St. 10) that uniformly expressed intense fluorescence of both YFP and AmCyan were screened. Swimming larvae at St. 58–59 (~4 months old) were incubated in a rearing solution containing 4 μM (Z)-4-Hydroxytamoxifen (4-OHT) for 48 h. Juvenile newts around 7 months old (1–2 months after metamorphosis) were subjected to surgical removal of both the lens and the neural retina under anesthesia and then allowed to recover for the study of retinal regeneration. (**b,c**) Representative of regenerated retinas at Stage L-2 (n = 4). mCherry fluorescence was observed in the central retina (box). The image is enlarged in (**c**). mCherry fluorescence was localized in both RPE cells and cells in the regenerated NR. In the regenerated NR, the mCherry+ cells composed a column structure similar to those observed in embryonic retinal development^7^,^8^,^9^. The column structure was comprised of all kinds of retinal neurons and glia. Interestingly, as in retinal development^9^, cell bodies of the horizontal cells were located distant from the body of the column. Note that in mCherry+ NR cells fluorescence of AmCyan was decreased (Cyan), indicating that Cre-mediated recombination successfully took place in parental RPE cells. Br: brain; ONL: outer nuclear layer; OPL: outer plexiform layer; INL: inner nuclear layer; IPL: inner plexiform layer; GCL: ganglion cell layer. Scale bars: 100 μm. (**d–f**) Contribution of the endogenous retinal stem/progenitor cells in the CMZ during retinal regeneration. Nuclei were counterstained with DAPI. Regenerating NR originating from the CMZ (arrow heads) had covered a large area of the RPE before the central RPE cells reached Stage E1 (right-hand box) to E2 (left-hand box) (n = 7). The images in these boxes are enlarged in (**e**,**f**) respectively. Dotted line in (**e**) shows Bruch’s membrane and that in (**f**) shows the outer margin of the regenerating NR. The arrow in (**f**) indicates a junction (called the Splayed-joint^1^) between the CMZ-originating (non-pigmented; left-hand) and RPE-originating regenerating NR (partially pigmented; right-hand). Scale bars: 100 μm. (**g**) Summary of results.

In the control condition with *C-shRNA*, retinal regeneration occurred normally (Fig. 1). We carried out retinectomy in ~7 months old juveniles (Fig. 1a). In the eyes at ~2 months post operation (po) when the retina had regenerated (Stage L-2), mCherry fluorescence was observed in both the NR and the RPE (Fig. 1b,c) as anticipated from our previous study^3^. However, in the NR, the mCherry+ area was restricted to the centre. It is known in growing eyes of juvenile newts that retinal stem/progenitor cells at the periphery of the NR (i.e., the ciliary marginal zone; CMZ), which is reserved from embryonic eye development, are still active, and their contribution to regeneration (or regrowth) of peripheral NR is very high compared to the adult^5^,^6^. In fact, also in our newts, a cell layer of the CMZ grew quickly toward the centre along the RPE upon retinectomy and met a pro-NR layer which formed slowly from the central RPE (Fig. 1d–f), eventually regenerating a large part of the NR (70–90% of its radius) while the remnant central NR was regenerated from the RPE (see the schematic in Fig. 1g). Participation of the peripheral RPE in the retinal regeneration was probably inhibited by association with the cell layer growing quickly from the CMZ. In the central NR, mCherry fluorescence was observed in all kinds of retinal neurons and glia (Fig. 1c). In particular cases, we recognized a column structure of NR cells (Fig. 1c and Supplementary Movie 1). Surprisingly, it resembled a column of descendants which originate from a single retinal progenitor cell in embryonic retinal development^7^,^8^,^9^. This finding is sufficient for us to believe that a single RPE-derived pro-NR cell is capable of producing one set of NR cells, being comparable to the retinal progenitor cell.

### Retinal regeneration in the Pax6 knockdown (Pax6 KD) condition

**Figure 2:**
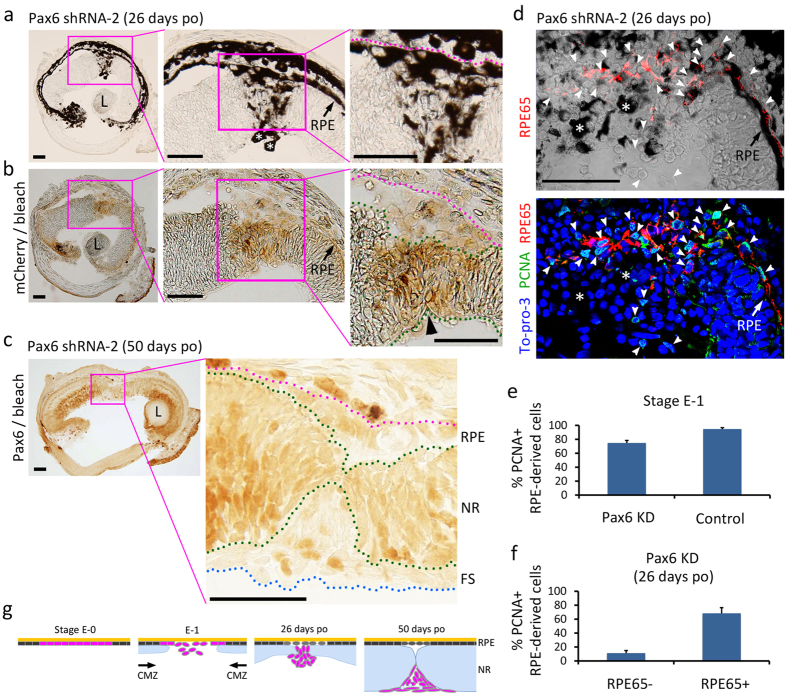
Retinal regeneration in Pax6 KD condition. (**a**) Regenerating retinas on 26 days post operation (op) (n = 5). (**b**) mCherry-immunolabeling of a neighbouring section of (**a**). The tissue was bleached. Lightly pigmented cells gathered in between the leading ends of CMZ-originating regenerating NR (CMZ-NR) (**a**). mCherry-immunoreactivity was localized in those cells (n = 4) (**b**). Pax6 expression in those cells was efficiently inhibited (see Fig. 3b). Arrowhead: a junction of leading ends of the CMZ-NR. Note that the cells in between Bruch’s membrane (dotted line in magenta) and the outer margin of the regenerating NR, i.e., in a region of prospective central RPE, had no mCherry-immunoreactivity (n = 3). (**c**) Regenerating retinas on 50 days po (n = 3). The section was labelled with Pax6 antibody and bleached. Pax6-less RPE-derived cells composed a fibrotic structure (FS) along the vitreal surface of the CMZ-NR (NR). Note that a small number of nuclei in the structure showed Pax6-mmunoreactivity. These cells probably migrated from the CMZ-NR (see Fig. 3). At this stage, Pax6-immunoreactivity became recognizable in the cells constructing a new central RPE. (**d**) RPE65− and PCNA-immunoreactivity in RPE-derived cells on 26 days po (n = 3). Arrowheads indicate PCNA+ nuclei. RPE65+ cells in a region of prospective central RPE seemed to have migrated from the surrounding RPE, and were mostly mitotically active (PCNA+). The cells on the margin of the surrounding RPE were also PCNA+. On the other hand, Pax6-less RPE-derived cells which gathered in between the leading ends of the CMZ-NR had lost RPE65-immunoreactivity and were rarely PCNA+. Note that green fluorescence surrounding migrating RPE cells and within the CMZ-NR were non-specific binding of PCNA antibody (see Methods). To-Pro-3: nucleus. L: Regenerated lens. Asterisk: Pigmented phagocyte^1^. Scale bars: 100 μm. (**e**) Comparison of the proportion of PCNA+ cells in all RPE-derived cells at Stage E-1 between the Pax6 KD (10 days po; n = 4) and the control (10 days po; n = 4) conditions. The mean value was significantly lower in Pax6 KD (Student’s *t*-test, *P* = 0.0017). (**f**) Proportion of PCNA+ cells in all RPE-derived cells without (RPE65−) or with RPE65− immunoreactivity (RPE65+) on 26 days po in the Pax6 KD condition (n = 3). The mean value was significantly lower in RPE65- (Student’s *t*-test, *P* = 0.0015). (**g**) Summary of results.

**Figure 3:**
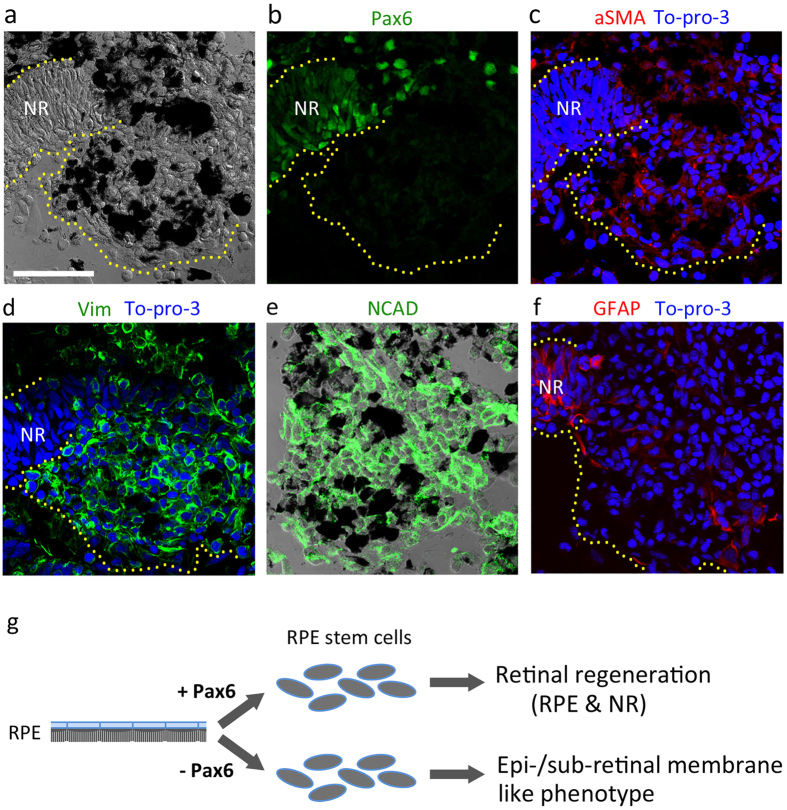
Appearance of epi-/sub-retinal membrane-like phenotype by Pax6 KD. (**a–f**) Representatives showing immunoreactions of Pax6-less RPE-derived cells on 26 days po (n = 5). NR: regenerating neural retina originating from the ciliary marginal zone. To-pro-3: nucleus. Scale bar (adapted to all panels): 100 μm. (**a**) Translucent image of a section. (**b,c**) Double labelling of the same section with Pax6 and alpha smooth muscle actin (aSMA) antibodies. (**d–f**) Labelling with Vimentin (Vim), N-cadherin (NCAD) and glial fibrillary acidic protein (GFAP) antibodies in different sections. Almost all of the Pax6-less RPE-derived cells in aggregates expressed aSMA, Vim and NCAD. On the other hand, GFAP immunoreactivity was localized in a small number of fibroblastic cells near the NR as well as in the NR, suggesting the migration of cells (possibly retinal progenitor cells) in the NR. Darkly pigmented cells in aggregates are ‘pigmented phagocytes’ that also appear in normal retinal regeneration^1^ (also see Fig. 2). Note that Pax6+ cells in aggregates (**b**) which seem to have migrated from NR, also expressed aSMA. (**g**) Schematic of conclusion.

In the Pax6 KD condition, obvious abnormalities appeared in the behavior of RPE cells as regeneration proceeded beyond Stage E1 (typically 10 days po) when RPE cells had become mesenchymal-like cells, which start to express Pax6 in normal retinal regeneration^3^,^10^ (Figs 2 and 3). In the eyes on 26 days po, when the thickness of a regenerating NR originating from the CMZ (CMZ-NR) had increased (Stage I-1 to I-2), lightly pigmented cells expressing mCherry, i.e., RPE-derived cells, gathered in between the leading ends of the CMZ-NR (Fig. 2a,b; see the schematic in Fig. 2g). However, unlike in the control condition, these cells were not integrated into one continuous regenerating NR. Immunohistochemistry demonstrated that Pax6 expression in those cells had been efficiently blocked (see Fig. 3b). On 50 days po when the CMZ-NR, in which the plexiform layers had just appeared (Stage I-3 to L-1), started fusing at its leading ends so that a hole in the centre of the regenerating NR closes, such Pax6-less (∆Pax6) RPE-derived cells had moved out to the vitreous cavity, creating a fibrous structure along the vitreal surface of the NR (Fig. 2c,g). We examined the proliferative activity of the RPE-derived cells (Fig. 2d–f). Cells in the centre of the RPE mostly re-entered the cell cycle by Stage E1 and proliferated (Fig. 2e), although activity decreased to ~80% of the control, and the cells derived from those RPE cells, i.e., ∆Pax6 RPE-derived cells, mostly exited the cell cycle by 26 days po (Fig. 2d,f). The ∆Pax6 RPE-derived cells at this stage had lost the RPE marker, RPE65 (Fig. 2d). Consequently, Pax6 KD hardly altered dedifferentiation (loss of original characteristics) and proliferation of RPE cells, but seriously impacted their transdifferentiation into the NR.

Regeneration of the central RPE was also observed later than 26 days po (Fig. 2). However, pigmented cells along or near Bruch’s membrane did not express mCherry (Fig. 2b). Instead, we observed Pax6 immunoreactivity in those cells on 50 days po (Fig. 2c), indicating that the central RPE originated from the cells in which Cre-mediated recombination did not take place. It is likely that most of those cells migrated from the margin of the surrounding RPE, whose participation in NR regeneration was inhibited by tissue that originated from the CMZ. In fact, on 26 days po, cells in the prospective central RPE exhibited RPE65 immunoreactivity as in the surrounding RPE, and those cells were cycling as were the cells in the margin of the surrounding RPE (Fig. 2d). Consequently, ∆Pax6 RPE-derived cells did not contribute to the renewal of RPE (Fig. 2g). Taken together, our results demonstrated that Pax6 expression in RPE-derived cells was essential for them to regenerate both the NR and the RPE.

### Appearance of epi-/sub-retinal membrane-like phenotype by Pax6 KD

We addressed whether impairment of retinal regeneration by Pax6 KD in RPE cells resulted in the appearance of symptoms of RPE-mediated retinal disorders. In human PVR, RPE cells undergo EMT, giving rise to myofibroblasts, which are a major component of the sub- and epi-retinal membranes^4^. In this study, using immunohistochemistry, we examined the expression of markers of RPE-derived myofibroblasts, such as alpha smooth muscle actin (aSMA), Vimentin and N-cadherin^4^,^11^, in ∆Pax6 RPE-derived cells (Fig. 3). Surprisingly, aggregates of ∆Pax6 RPE-derived cells, which had migrated from their original place on Bruch’s membrane and gathered in between the leading ends of the CMZ-NR, expressed aSMA, Vimentin and N-cadherin uniformly (Fig. 3c–e). We further examined the expression of glial fibrillary acidic protein (GFAP), another marker of epi- and sub-retinal membranes^4^. GFAP immunoreactivity was localized in fibroblastic cells near the leading ends of the CMZ-NR (Fig. 3f). These cells may have migrated from regenerating NR. GFAP is known to be expressed in both retinal progenitor cells and Müller glia cells^12^. In PVR, Müller cells in an injured NR also contribute to the epi- and sub-retinal membranes^4^. Taken together, Pax6 KD in RPE-derived cells resulted in a structure with a similar phenotype to the epi- and sub-retinal membranes (Fig. 3g).

## Discussion

In this study, we successfully hindered RPE-originating retinal regeneration in the newt by knocking down Pax6 in RPE-derived cells. We must note here that in the juvenile eyes examined, the contribution of the CMZ to retinal regeneration was so high that the area in which RPE-originating retinal regeneration took place was restricted to the central retina. In the Pax6 KD condition, RPE-derived mesenchymal-like cells were able to migrate and proliferate but eventually differentiated into cells like myofibroblasts, suggesting that the newt RPE cells underwent EMT. Furthermore, cellular components of the resulting structure were similar to those of epi- and sub-retinal membranes. In conclusion, inhibition of the retinal regeneration resulted in the appearance of symptoms of RPE-mediated retinal disorders like PVR. Possibly, adult newts may have invented the ability of retinal regeneration by revising the program underlying EMT of RPE cells upon retinal trauma, so that the fate of RPESCs is directed toward the retinal regeneration. Clearly, in the newt, Pax6 is an essential player for this process with a novel function enabling RPESCs to self-organize into two progenitor layers (epithelium) which eventually regenerate the NR and the RPE.

In a previous study, we demonstrated that Pax6 KD in embryonic development resulted in a missing eye in the newt, suggesting that the function of Pax6 as a master regulator of eye morphogenesis is also conserved in this animal^3^. In the current study, we examined the function of Pax6 in a different context, i.e., retinal regeneration from terminally differentiated RPE cells, that is unique to this animal. However, comparison with eye development may be helpful to understand Pax6 function in this system. In our previous study, we proposed that RPESCs might substantially correspond to the cells in the early optic vesicle although RPESCs preserved their original characteristics^3^. Furthermore, we hypothesized, as an analogy to optic cup formation, that Pax6 might have a role in the fate decision of RPESCs to differentiate either the NR or the RPE as the cells formed two progenitor layers^10^. However, in the current study, we demonstrated that in the Pax6 KD condition, RPE-derived cells differentiated into neither the NR nor the RPE, but rather into myofibroblast-like cells. These findings suggest that Pax6 is essential for RPE cells to be reprogrammed into normal RPESCs which have the potency to differentiate into both the NR and the RPE (Fig. 3g). In speculation, RPE cells might substantially dedifferentiate to the state near the eye field from which the optic vesicle is formed. Given this, the time point at which Pax6 functioned at first was earlier than anticipated, and therefore the previous hypothesis was not evaluated in this study. However, we know that once RPESCs are generated, they undergo regulation of Pax6 and are sorted into two cell populations^3^. One population in which Pax6 is up-regulated forms the pro-NR layer, while the other population forms the pro-RPE layer^3^. Therefore, the function of Pax6 in cell fate decision is reasonable. Note that in this stage a mesenchymal to epithelial transition (MET)-like event takes place in both progenitor layers. Our Pax6 KD system can target RPESCs, and therefore we will pursue this issue in the future.

It would be difficult to understand by comparison with eye development how normal reprogramming by Pax6 prevents RPE cells from transforming into myofibroblast-like cells. This issue would be of mature RPE cells. Pax6 may function even as a key factor that revises a default program which is booted in mature RPE cells after retinal injury and leads them to EMT. It must be noted here that Pax6 may be expressed in human RPESCs which give rise to myofibroblast cells in PVR^3^,^4^. In the newt, something might have happened in RPE cells during evolution so that Pax6 can work properly for retinal regeneration while inhibiting EMT. Further understanding of how Pax6 works in reprogramming RPE cells in the adult newt in comparison with the homologous system in humans is necessary not only to uncover the changes that occurred in the newt during evolution but also to unlock the potency of *in vivo* retinal regeneration from RPE cells in humans. These findings would lead, in the future, to a novel clinical treatment of RPE-mediated retinal disorders that inhibits the EMT of RPE cells while promoting retinal regeneration in the eyes of patients.

## Methods

All methods were carried out in accordance with Regulations on the Handling of Animal Experiments in University of Tsukuba (RHAEUT). All experimental protocols were approved by Committee for Animal Experiments in University of Tsukuba (CAEUT).

### Animals

Toride-Imori, a race of the Japanese fire-bellied newt *C. pyrrhogaster*, was used for this study^13^. Fertilized eggs were obtained from adult animals (total body length: male, ~9 cm; female, 11–12 cm)^13^. Developmental stages were determined according to previous criteria^13^.

### Transgenesis for RPE-targeted Pax6 knockdown

To block the expression of Pax6 in both mature RPE cells and cells originating from those RPE cells after retinectomy, two plasmids, pRPE65 > CreER^T2^-CAGGs > YFP (I-SceI) and pCAGGs > [AmCyan]mCherry-shRNA (I-SceI), were constructed based on cpRPE65-mcherry01^14^ and pCAGGs-mCherry-shRNA (Sce)^3^ using standard molecular cloning procedures (Supplementary Fig. 1a). In pRPE65 > CreER^T2^-CAGGs > YFP (I-SceI), the *C. pyrrhogaster* RPE65 promoter, which is specifically activated in RPE cells as they reach terminal differentiation^14^, was used to drive the expression of an inactive form of Cre recombinase CreER^T2^, and a fluorescent protein YFP, which is expressed under the control of a ubiquitous promoter CAGGs^13^, was used to monitor the transgenic state. In pCAGGs > [AmCyan]mCherry-shRNA (I-SceI), a gene of the fluorescent protein AmCyan (Z2440N; Takara Bio, Shiga, Japan) was flanked by *loxP* sites, and a DNA sequence encoding *Pax6 shRNA-2* or control shRNA (*C-shRNA*) was inserted between the stop codon and the polyadenylation terminal in a gene of the other fluorescent protein mCherry. In the presence of the activated Cre, AmCyan expression, which is driven by CAGGs and was monitored to evaluate the transgenic state, is switched to mCherry-shRNA expression. The design and specificity of shRNA have been described in our previous paper^3^. In both plasmids, the transgene cassette was flanked by I-SceI meganuclease recognition sites. The chicken β-globin HS4 2× core insulator was inserted into the cassette to minimize possible *cis* interactions within the cassette and between the cassette and functional elements on the chromosome. The HS4 insulator was a kind gift from Dr. Gary Felsenfeld (National Institute of Health, Bethesda, MD, USA).

Transgenesis was carried out using the I-SceI protocol^13^ (Supplementary Fig. 1b). Two plasmids (described above) and I-SceI enzyme (catalogue #R06945; New England Biolabs, Tokyo, Japan) were co-injected into one-cell stage embryos. Components of the injected solution (4 nl per embryo) were as follows: pRPE65 > CreER^T2^-CAGGs > YFP (I-SceI), 40 ng μl^−1^; pCAGGs > [AmCyan]mCherry-shRNA (I-SceI), 40 ng μl^−1^; I-SceI, 1 U μl^−1^; I-SceI buffer (New England Biolabs), 1X; phenol red, 0.01%. The injected embryos were incubated until the 4-cell stage at 12 °C overnight. When the embryos reached the blastula stage (St. 10), the animals that expressed both YFP and AmCyan in their entire body almost evenly were screened under a fluorescence dissecting microscope (M165 FC; Leica Microsystems, Wetzlar, Germany) (Supplementary Fig. 1c). They were reared until the swimming larva stage (St. 58–59) at 22 °C. Leaky expression of mCherry was not detected during development (Supplementary Fig. 1c). To induce Cre-mediated recombination, when swimming larvae reached St. 59 (just before metamorphosis), they were transferred into 0.1X Holtfreter’s solution^13^ (pH 7.4) containing 4 μM 4-OHT [(Z)-4-hydroxytamoxifen; Sigma-Aldrich, MO 63103, USA] and 1% (v/v) dimethylsulfoxide (DMSO), and incubated for 48 h at 22 °C in the dark (the solution was exchanged to a fresh solution after 24 h incubation) (Supplementary Fig. 2a). For the control, animals were incubated in 0.1X Holtfreter’s solution containing 1% (v/v) DMSO only. After 4-OHT treatment, animals were transferred into 0.1X Holtfreter’s solution at 22 °C until metamorphosis. Metamorphosed juveniles were transferred into moist chambers and reared until surgery.

### Anesthesia

Juvenile newts were anesthetized in water containing 0.05% (v/v) FA-100 (4-allyl-2-methoxyphenol; DS Pharma Animal Health, Osaka, Japan) for 15 min at 20 °C in the dark. Anesthetized animals were rinsed in distilled water and air dried on moist paper towels until surgery.

### Retinectomy

Juvenile newts around 7 months old (1–2 months after metamorphosis; total body length: 3.5–4.0 cm) were subjected to surgical removal of both the lens and the neural retina under anesthesia. Animals were placed under a dissecting microscope (M165 FC). The dorsal half of the eye was cut open along the corneal-scleral junction, and the neural retina was carefully removed together with the lens^1^. After surgery, animals were allowed to recover and kept in moist chambers at 20 °C until experiments.

### Tissue preparation

Eyeballs were carefully removed from animals under anesthesia. A fragment of the dorsal eyelid was left on the eyeballs. For direct observation of reporter fluorescence (mCherry, Cyan, and YFP), eyeballs were fixed in phosphate-buffered saline (PBS; pH 7.4) containing 4% (w/v) paraformaldehyde and 0.25% (v/v) glutaraldehyde for 6 h at 4 °C (Fig. 1b,c and Supplementary Fig. 1c). Note that this fixation condition was too strong to detect gene expression by immunohistochemistry. For immunohistochemistry, eyeballs were fixed in modified Zamboni’s solution [2% (w/v) paraformaldehyde and 0.2% (w/v) picric acid in PBS (pH 7.4)] for 6 h at 4 °C (Fig. 2, Fig. 3 and Supplementary Figs. 2 and 3). Note that in this condition, reporter fluorescence was diminished, while red autofluorescence sometimes appeared along the outer segments of photoreceptors and red blood cells in the choroid (Supplementary Fig. 3b). Fixed eyeballs were thoroughly rinsed in PBS at 4 °C (5 min × 3, 10 min × 3, 30 min × 3, 1 h × 3, and overnight), transferred to 30% sucrose in PBS at 4 °C and incubated until the eyeballs were completely submerged (7–9 h). They were embedded into Tissue-Tek O.C.T compound (4583; Sakura Finetek USA, CA 90501, USA) and cryosectioned at ~20 μm thickness along the dorsoventral axis^1^.

### Immunohistochemistry

Primary antibodies used were as follows: mouse monoclonal anti-Pax6 antibody (1:200; AD2.38, sc-32766; Santa Cruz Biotechnology, TX 75220, USA), anti-RPE65 antibody (1:500; MAB5428; EMD Millipore, CA 92590, USA), anti-Vimentin antibody [Vim 3B4] (1:250; ab28028; Abcam, Tokyo 103–0012, Japan) and Cy3 conjugated anti-GFAP antibody (1:600; G-A-5-Cy3; C9205; Sigma-Aldrich); rabbit polyclonal anti-RFP antibody (for mCherry; 1:200–500; 600-401-379; Rockland Immunochemicals, PA 19468, USA), anti-aSMA antibody (1:500; ab137734; Abcam) and anti-N Cadherin (1:400; ab12221; Abcam); human PCNA autoantibody^1^ (1:1000). Secondary antibodies were as follows: Alexa 488-conjugated goat anti-mouse IgG (H + L) antibody (1:500; A11001; Thermo Fisher Scientific, Yokohama, Japan), anti-rabbit IgG (H + L) antibody (1:500; A11034; Thermo Fisher Scientific) and anti-human IgG antibody (1:1,000; A11013; Thermo Fisher Scientific); tetramethylrhodamine-conjugated goat anti-mouse IgG antibody (1:250; T2762; Thermo Fisher Scientific); Rhodamine (TRITC)-conjugated affiniPure goat anti-rabbit IgG (H + L) antibody (1:500; Code: 111-025-003; Jackson ImmunoResearch Laboratories, PA 19390, USA); biotinylated goat anti-mouse IgG antibody (1:250; BA-9200; Vector Laboratories, CA 94010, USA) and anti-rabbit IgG (1:250; BA-1000; Vector Laboratories).

For immunofluorescence labeling^3^, eyeball sections were washed (PBS, 15 min → 0.1% Triton X-100 in PBS, 15 min → PBS, 15 min), incubated with blocking solution [2% normal goat serum (S-1000; Vector Laboratories), 5% bovine serum albumin (A3294-50G; Sigma-Aldrich) and 0.1% Triton X-100 in PBS] for 2 h, and then incubated with primary antibody diluted in blocking solution overnight at 4 °C. The sections were washed and then incubated with secondary antibody diluted in blocking solution for 4 h. To minimize autofluorescence, eyeball sections were sometimes treated with 0.3% H_2_O_2_ in 50% methanol diluted in PBS for 20 min before they were incubated with blocking solution (Fig. 3). For immunoperoxidase labeling^3^, eyeball sections were washed, treated with 0.3% H_2_O_2_ in methanol for 20 min, washed, incubated in blocking solution mixed with Avidin D (1:50; Avidin/Biotin Blocking kit; SP-2001; Vector Laboratories) for 2 h, washed twice in PBS, and then incubated in primary antibody diluted with blocking solution mixed with Biotin (1:50; Avidin/Biotin Blocking kit) overnight at 4 °C. The sections were washed, incubated with biotinylated secondary antibody diluted in blocking solution for 4 h, washed, incubated in a mixture of Avidin and Biotin Complex (1:50 each; Vectastain ABC Elite kit; PK-6100; Vector Laboratories) for 2 h, washed, and then treated with DAB solution (DAB substrate kit; SK-4100; Vector Laboratories). After labeling, tissues were washed and sometimes bleached^3^ (Fig. 2b,c and Supplementary Fig. 2b). They were counterstained to visualize nuclei with either DAPI (4,6-diamidino-2-phenylindole; 1:25,000; D1306; Thermo Fisher Scientific) or TO-PRO-3 Iodide (1:10,000; T3605; Thermo Fisher Scientific), and finally mounted with 90% glycerol in PBS or Vectashield mounting medium (H-1000; Vector Laboratories).

### Image analysis

Transgenic newts were monitored under a fluorescence dissecting microscope (M165 FC) which was installed with filter sets specific to AmCyan (CFP; Exciter: 436/20 nm; Emitter: 480/40 nm; Leica Microsystems), YFP (YFP; Exciter: 500/20 nm; Emitter: 535/30 nm; Leica Microsystems) and mCherry (Exciter: XF1044, 575DF25; Emitter: XF3402, 645OM75; Opto Science, Tokyo, Japan). Images were taken by a digital camera system (EOS Kiss x7i; Canon, Tokyo, Japan) attached to the microscope. Eyeball sections were examined on a fluorescence microscope (BX50; Olympus, Tokyo, Japan) which was also installed with the same filter sets as well as standard filter sets for EGFP, Texas red and DAPI. Their images were taken by a charge-coupled device camera system (DP73; cellSens Standard 1.6; Olympus) attached to this microscope (BX50; Olympus) or by a confocal microscope system (LSM510; LSM 5.0 Image Browser software; Carl Zeiss, Germany). Images were analyzed with software for the image acquisition systems and in Photoshop CS5 Extended (Adobe Systems, San Jose, CA). Figures and panels were prepared using Photoshop CS5 Extended. Images, brightness, contrast and sharpness were adjusted according to the journal’s guidelines.

### Cell counting and statistics

To count the number of PCNA+ RPE-derived cells (Fig. 2d–f), eyeball sections were double-labelled with antibodies against PCNA and RPE65, and stained with To-pro-3 to visualize cell nuclei (see above). Sections were carefully examined for their immunoreactivities by confocal microscopy (see above). PCNA-immunoreactive nuclei were counted in all nuclei (To-pro-3 stained) of RPE-derived cells on 10 days [Stage E-1; these cells were still RPE65+ in both Pax6 KD and control conditions (Fig. 2e)], and in all nuclei of RPE-derived cell aggregates in between the leading ends of CMZ-NR on 26 days po [these cells were either RPE65− or RPE65+ (Fig. 2f)]. Of note, in PCNA immunohistochemistry under the current conditions using juvenile eyes, non-specific binding of primary antibody sometimes occurred in the intercellular space within regrowing NR from CMZ (CMZ-NR) and in the extracellular matrix surrounding RPE cells migrating from the ends of regrowing RPE. For statistics, a section containing aggregates of RPE-derived cells was selected per eyeball at random from those around the centre of the eyeball, and the cell count data from the section was used as representative data. Data were collected from at least three eyeballs in both the control and test conditions, and presented as the mean ± SE. Statistical differences between the control and test was evaluated by a Student’s *t*-test.

## Additional Information

**How to cite this article**: Casco-Robles, M. M. *et al.* Turning the fate of reprogramming cells from retinal disorder to regeneration by Pax6 in newts. *Sci. Rep.* **6**, 33761; doi: 10.1038/srep33761 (2016).

## Supplementary Material

Supplementary Information

Supplementary Movie 1x
