# Supplementary material for: Turning the fate of reprogramming cells from retinal disorder to regeneration by Pax6 in newts
Source: Sci Rep. 2016 Sep 19;6:33761. doi: 10.1038/srep33761 (PMC5027390; doi:10.1038/srep33761)
Supplement: Supplementary Information [file srep33761-s1.pdf]

Supplementary Information

Turning the fate of reprogramming cells from retinal disorder to regeneration by Pax6 in newts

Martin Miguel Casco-Robles[1], Md Rafiqul Islam[2], Wataru Inami[3], Hibiki Vincent Tanaka[3], Ailidana Kunahong[3], Hirofumi Yasumuro[3], Shiori Hanzawa[3], Roman Martin Casco-Robles[3], Fubito Toyama[4], Fumiaki Maruo[1] and Chikafumi Chiba[1]

[1] Faculty of Life and Environmental Sciences, University of Tsukuba, Tennodai 1-1-1, Tsukuba, Ibaraki 305-8572, Japan

[2] Department of Genetic Engineering and Biotechnology, University of Chittagong, Chittagong-4331, Bangladesh

[3] Graduate School of Life and Environmental Sciences, University of Tsukuba, Tennodai 1-1-1, Tsukuba, Ibaraki 305-8572, Japan

[4] Graduate School of Engineering, Utsunomiya University, Yoto 7-1-2, Utsunomiya, Tochigi 321-8585, Japan

Correspondence and requests for materials should be addressed to C.C. (email: chichiba@biol.tsukuba.ac.jp) or to M.M.C.-R. (email: casco.m.m.gm@u.tsukuba.ac.jp).

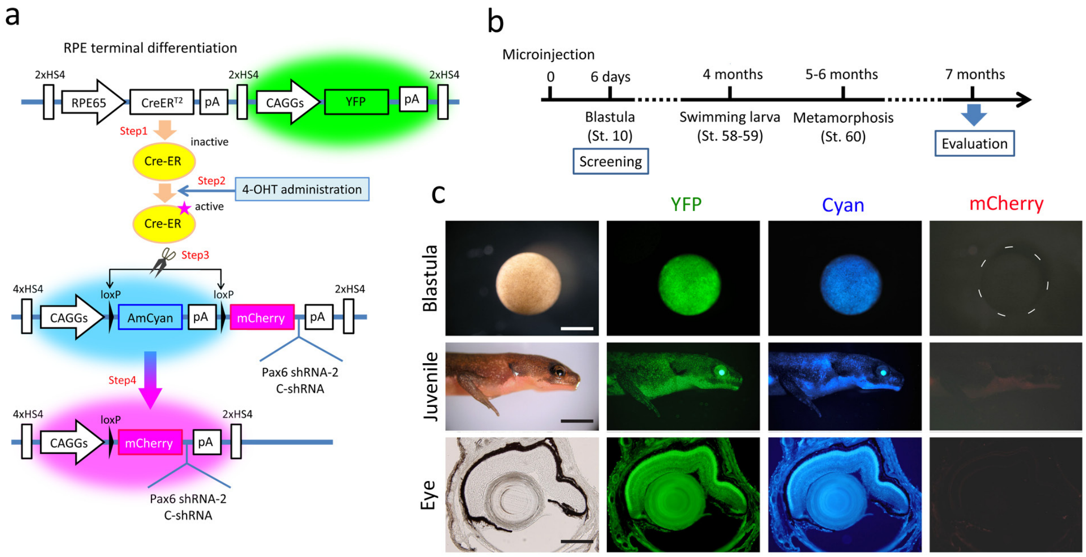

Supplementary Figure 1| Conditional RPE-targeted gene knockdown system in the Japanese fire bellied newt, *Cynops pyrrhogaster*. (a), Construct design. We designed a Cre driver construct (RPE65>CreER^{T2}-CAGGs>YFP) and a loxP reporter/shRNA construct (CAGGs>[AmCyan]mCherry-shRNA) to create, using the I-SceI protocol[13], transgenic newts that specifically express shRNA as well as a fluorescent reporter protein mCherry in both mature RPE cells and cells that originate from those RPE cells after retinectomy. Transgenic newts carrying both constructs should exhibit both YFP and AmCyan fluorescence throughout their body. When RPE cells reach their terminal differentiation, the inactive form of Cre (CreER^{T2}) would be expressed under the control of the *RPE65* promoter[14] (Step 1). When Cre is activated by administration of (Z)-4-Hydroxytamoxifen (4-OHT; Step 2), the *AmCyan* gene in the loxP reporter/shRNA construct is removed and the *mCherry* gene containing a shRNA cassette [carrying *Pax6 shRNA-2* or control shRNA (*C-shRNA*)] is ligated to a ubiquitous promoter *CAGGs*[13] (Step 3), making the RPE cells express mCherry-shRNA (Step 4). If this system works, all of the cells originating from RPE cells after retinectomy should also express mCherry-shRNA. Note that the I-SceI recognition sequence was placed on both sides of the transgene cassettes. HS4: chicken beta-globin insulator. (b,c), Evaluation of this system with *C-shRNA*. (b), Time schedule of experiments. One-cell stage embryos were microinjected with a solution containing two constructs and I-SceI meganuclease. On the sixth day after injection, blastula embryos (St. 10) that uniformly expressed intense fluorescence of both YFP and AmCyan were screened. The expression pattern of transgenes was examined in juvenile newts around 7 months old (1-2 months after metamorphosis). (c), Representatives of the patterns of YFP, AmCyan (Cyan) and mCherry fluorescence in blastula embryos (n=93), juveniles (n=69) and eyes of juveniles (n=4). Animals exhibited both YFP and AmCyan fluorescence ubiquitously. Leaky expression of mCherry was not detected. No abnormalities in eye development or morphology were observed. Scale bars: 1 mm (blastula), 5 mm (juvenile), 200 μm (eye).

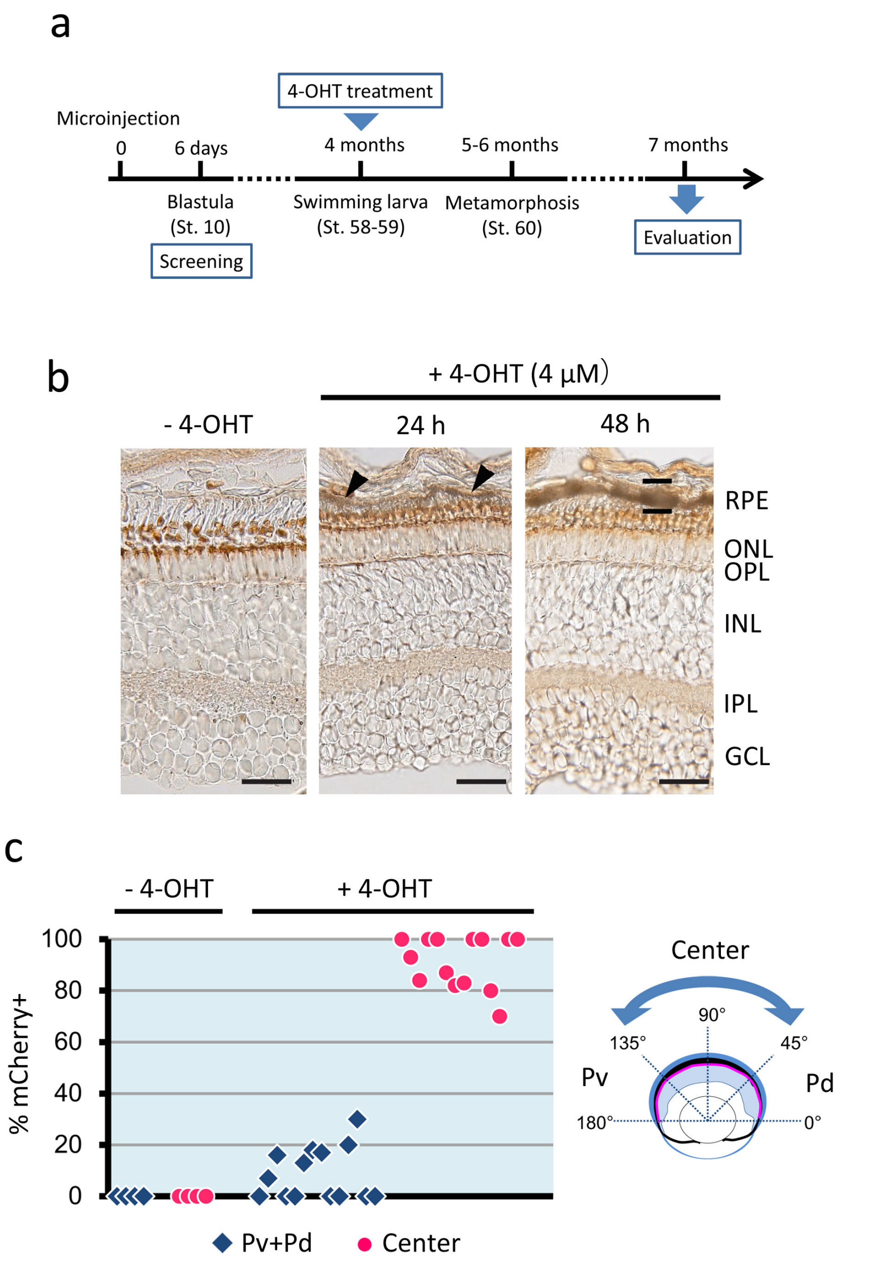

Supplementary Figure 2| Induction of Cre-mediated recombination in mature RPE cells. (a), Time schedule of experiments. Microinjection and screening were explained as shown in Supplementary Fig. 1. To induce Cre-mediated recombination in RPE cells, swimming larvae at St. 58-59 (~4 months old) were incubated in a rearing solution containing (Z)-4-Hydroxytamoxifen (4-OHT). In these stages, almost all RPE cells, except for those at the peripheral margin of the RPE, had matured while expressing RPE65[3]. The expression pattern of transgenes was examined in juvenile newts around 7 months old (1-2 months after metamorphosis). (b), Optimization of 4-OHT administration conditions. Eye sections of juveniles carrying the mCherry-C-shRNA construct were labelled with mCherry antibody and bleached. Expression of mCherry in RPE cells became detectable after a swimming larva was

incubated in 4 µM 4-OHT for 24 h (arrowheads in 24h). Expression increased drastically as the incubation period was doubled using fresh 4-OHT solution (see the signal in between horizontal bars in 48 h). Note that longer incubation with 4 µM 4-OHT influenced survival rate. -4-OHT: control without 4-OHT administration. ONL: outer nuclear layer; OPL: outer plexiform layer; INL: inner nuclear layer; IPL: inner plexiform layer; GCL: ganglion cell layer. Scale bars: 50 µm. (c), Recombination efficiency. It was evaluated by counting mCherry+ cells along the RPE. For this analysis, a total of 13 eyeballs were harvested from different juveniles carrying the mCherry-C-shRNA construct and treated with 4 µM 4-OHT for 48 h in the swimming larva stage. Eyeballs were sliced through the dorsoventral axis, and a section containing the centre of the eye was selected for each eyeball and processed as in (b). In these 13 representative sections, mCherry expression along the RPE was examined. In the centre (45°-135°) of the retina (see the inset schematic), more than 70% of RPE cells were mCherry+, while at the periphery of the retina (Pv+Pd), less than 30% of RPE cells were mCherry+. -4-OHT: control without 4-OHT administration (biological replicates = 4).

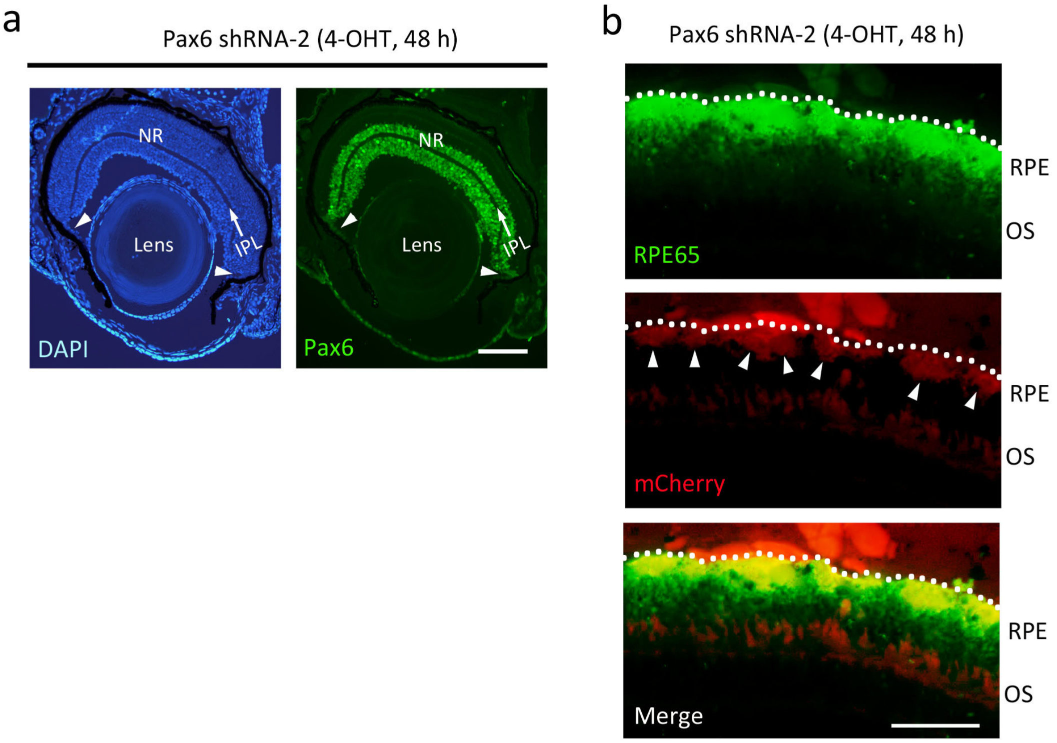

Supplementary Figure 3| Induction of mCherry-Pax6 shRNA-2 expression in mature RPE cells. *Pax6 shRNA-2* has proved to effectively knockdown Pax6 expression in this species[3]. Animals carrying the mCherry-Pax6 shRNA-2 construct were treated with 4 µM 4-OHT for 48 h in the swimming larva stage. This treatment did not affect normal eye development and Pax6 expression in the eye tissues. (d), Representative showing eye morphology and Pax6 immunoreactivity in juveniles (n=4). Note that in this fixation condition with modified Zamboni's solution, fluorescence of YFP, AmCyan and mCherry almost disappeared (see Methods). NR: neural retina. Arrowheads: CMZ. DAPI: nucleus. Scale bar: 200 µm. (e), Representative showing mCherry expression in mature RPE cells (n=6). mCherry immunoreactivity was detected in mature RPE cells characterized by their microvilli and RPE65 immunoreactivity (arrowheads). Dotted line shows Bruch's membrane. Note that in this fixation condition bright red autofluorescence was observed in the outer segments of photoreceptors (OS) and blood cells in the choroid (compare to Supplementary Fig. 2b). Scale bar: 50 µm.

Supplementary Movie 1| RPE originating retinal cells (mCherry+) in a regenerated retina in control condition.
